# 18F-FDG PET Radiomics as Predictor of Treatment Response in Oesophageal Cancer: A Systematic Review and Meta-Analysis

**DOI:** 10.3389/fonc.2022.861638

**Published:** 2022-03-15

**Authors:** Letizia Deantonio, Maria Luisa Garo, Gaetano Paone, Maria Carla Valli, Stefano Cappio, Davide La Regina, Marco Cefali, Maria Celeste Palmarocchi, Alberto Vannelli, Sara De Dosso

**Affiliations:** ^1^ Radiation Oncology Clinic, Oncology Institute of Southern Switzerland (IOSI), Bellinzona, Switzerland; ^2^ University of Southern Switzerland, Faculty of Biomedical Sciences, Lugano, Switzerland; ^3^ Mathsly Research, Brescia, Italy; ^4^ Clinic for Nuclear Medicine and Molecular Imaging, Imaging Institute of Southern Switzerland, Ente Ospedaliero Cantonale, Bellinzona, Switzerland; ^5^ Clinic for Radiology, Imaging Institute of Southern Switzerland, Ente Ospedaliero Cantonale, Bellinzona, Switzerland; ^6^ Department of Surgery, Ente Ospedaliero Cantonale, Bellinzona, Switzerland; ^7^ Department of Medical Oncology, Oncology Institute of Southern Switzerland (IOSI), Bellinzona, Switzerland; ^8^ Department of General Surgery, Ospedale Valduce, Como, Italy

**Keywords:** oesophageal cancer, radiomics, 18F-FDG PET, complete clinical response, pathological complete response, neoadjuvant chemoradiotherapy

## Abstract

The best treatment strategy for oesophageal cancer patients achieving a complete clinical response after neoadjuvant chemoradiation is a burning topic. The available diagnostic tools, such as 18F-FDG PET/CT performed routinely, cannot accurately evaluate the presence or absence of the residual tumour. The emerging field of radiomics may encounter the critical challenge of personalised treatment. Radiomics is based on medical image analysis, executed by extracting information from many image features; it has been shown to provide valuable information for predicting treatment responses in oesophageal cancer. This systematic review with a meta-analysis aims to provide current evidence of 18F-FDG PET-based radiomics in predicting response treatments following neoadjuvant chemoradiotherapy in oesophageal cancer. A comprehensive literature review identified 1160 studies, of which five were finally included in the study. Our findings provided that pooled Area Under the Curve (AUC) of the five selected studies was relatively high at 0.821 (95% CI: 0.737–0.904) and not influenced by the sample size of the studies. Radiomics models exhibited a good performance in predicting pathological complete responses (pCRs). This review further strengthens the great potential of 18F-FDG PET-based radiomics to predict pCRs in oesophageal cancer patients who underwent neoadjuvant chemoradiotherapy. Additionally, our review imparts additional support to prospective studies on 18F-FDG PET radiomics for a tailored treatment strategy of oesophageal cancer patients.

**Systematic Review Registration:**

https://www.crd.york.ac.uk/prospero/, identifier CRD42021274636.

## Introduction

Globally, oesophageal carcinoma is the seventh most frequently diagnosed cancer and sixth leading cause of cancer-related death (1). In 2020, about 604.100 new cases were estimated, resulting in nearly 544.000 deaths (2). To date, neoadjuvant chemoradiotherapy followed by surgery is considered the standard care for patients with resectable locally advanced oesophageal and gastro-oesophageal junction cancer, and 25–42% of patients achieved a pathological complete response (pCR) following such treatment (3, 4). More in detail, between 20 and 30% of patients with adenocarcinoma and 40% with squamous cell histology are expected to achieve a pCR following a multimodal therapy (4)

Despite the survival benefits of this combined approach, oesophagectomy is considered a highly invasive procedure with a significant rate of morbidity and mortality, potentially affecting long-term quality of life. Consequently, the active surveillance strategy in case of clinical complete response (cCR) following chemoradiotherapy is a debatable topic (5–7). This strategy appears appealing and should be based on the risk of relapse, quality of life, and morbidity due to the multimodality treatment approach, along with survival expectancy. Consequently, the reliability of non-operative diagnostic tools, which identify complete clinical response, is crucial. However, one of the practical obstacles in selecting patients for immediate surgery or close observation is poor ability to predict a pCR before surgery with the currently available imaging tools.

On the other hand, persistent disease after chemoradiotherapy is usually associated with poorer long-term prognosis, which may suggest more aggressive and resistant tumour biology requiring an immediate and aggressive surgical approach. The early identification of tumours not responding to chemoradiotherapy is clearly another significant area for future investigation on the optimal timing of the treatment sequence.

Endoscopy/endoscopic ultrasound and 18F-fluorodeoxyglucose positron emission tomography/computed tomography (18F-FDG PET/CT) are the current diagnostic tools for baseline staging as well as the evaluation of treatment responses. Although endoscopy and ultrasound have an accuracy assessment of around 70%, and 18-FDG PET/CT identified a complete response in 71–88% of cases, no current procedure can accurately predict the treatment response (8).

To perform a personalised approach of combined treatment or surveillance after neoadjuvant radiochemotherapy, an accurate patient stratification is the main issue. In this regard, physicians perceive radiomics with great interest, and the opportunity to offer a personalised treatment to our patients seems to be closer than before.

Radiomics involves the automatic extraction of a range of quantitative features from radiologic images (i.e., 18F-FDG PET/CT, CT, and magnetic resonance imaging (MRI)) to arrive at a comprehensive quantification of tumour phenotypes for the prediction of the treatment response and outcome (9). This emerging field is rapidly gaining scientific interest for its potential clinical implications (9–11). In this regard, the concept of precision medicine could be supported by radiomics. Its findings can be applied to individual patients, although the relationship between radiomics and outcomes are derived from populations. Furthermore, radiomics analysis commits to increase accuracy in diagnosis, evaluation of prognosis, and prediction of therapy response (12).

Preliminary data for oesophageal and gastro-oesophageal cancer suggest a potential for radiomics approaches in improving patient stratification for therapy (13).

To date, the published studies are based on several radiologic images (i.e 18F-FDG PET, CT, and MRI) and systematic reviews and meta-analysis based their conclusions on radiomics findings of both morphologic and metabolic diagnostic images (14).

18F-FDG PET-based radiomics seems promising for the management of oesophageal cancer patients concerning the prediction of the treatment response in addition to orienting tailored treatments (15). However, the power of the current 18F-FDG PET/CT radiomics algorithms to predict a pCR in oesophageal cancer in patients who underwent neoadjuvant chemoradiotherapy is an unmet need. The present systematic review and meta-analysis aimed to collect the current evidence of 18F-FDG PET-based radiomics in predicting the response treatment following neoadjuvant chemoradiotherapy in oesophageal cancer. The findings can lead to build future PET-based radiomics prospective trials for predicting pCR in oesophageal and gastro-oesophageal junction cancer.

## Methods

Preferred Reporting Items for Systematic Reviews and Meta-Analysis (PRISMA) guidelines were employed in conducting this study (16). The methodology was previously registered in the International Prospective Register of Systematic Reviews (PROSPERO) database under the protocol number CRD42021274636.

### Data Sources and Search Strategy

A comprehensive search strategy, used on PubMed, Scopus, Web of Science, and EMBASE to identify all relevant studies irrespective of language or publication status, was performed until 15 November 2021. Duplicates were manually removed. After a comprehensive selection process, the reference lists of all the identified studies were checked.

### PubMed Search Strategy

The search strategy was (Oesophageal OR esophageal OR oesophagogastric OR esophagogastric OR gastro-oesophageal OR gastro-esophageal) AND (cancer OR neoplasia) AND (radiomics OR radiomics features OR radiomic) AND (response OR remission OR outcome OR prognostic OR predictive OR predicting OR prediction)

### Inclusion Criteria and Study Selection

Studies were included if they strictly met the following criteria: 1) Patients with oesophageal and gastro-oesophageal cancer who had received neoadjuvant chemoradiation; 2) 18F-FDG PET/CT imaging was performed; 3) Radiomics was used to predict a pCR; 4) Area Under the Curve (AUC) was determined; 5) Any models/algorithm applied to predict the pathological response.

Studies that did not report results in AUC, accuracy, sensitivity, and specificity, lacked proof of validation, or had insufficient detail regarding algorithm development and extraction of diagnostic accuracy were excluded.

### Implementation of Search

Two reviewers independently screened the identified articles based on their titles and abstracts (LD and SDD), which were considered in constructing a list of all potentially relevant papers. The full-text versions of potentially eligible studies were assessed against the eligibility criteria. The authors planned to solve disagreements concerning study selection or quality assessment by consensus or discussion with a third member of the review team (AV) and reported this in the final review. However, no disagreement was present, and consequently, the kappa statistic was not determined.

### Outcome Measures and Data Extraction

The primary endpoint was set as the highest AUC in the validation set (training set). When external validation was not present, we chose internal validation results. If the internal validation was not reported, the result from the training set was chosen. In the absence of the AUC, the C-index was used.

Two authors (LD and SDD) independently extracted the following information:

1. General study characteristics (authors, year, country)
2. Study population (source of data and sample size)
3. Clinical outcomes (pathological response)
4. Treatment schedule
5. Algorithm used for the outcome prediction
6. Dimensionality reduction methods
7. Results: highest AUC and standard error

If the standard error was not indicated, we determined it through Hanley and McNeil’s formula (17). On the other hand, we determine the standard error using the conventional procedure if the standard deviation was reported.

### Quality Assessment

Two authors (LD and SDD) assessed the study quality through the Radiomics Quality Score (RQS) (18), ranging from a minimum score of -5 to a maximum score of 36 points.

### Risk of Bias

The researchers planned to assess the risk of bias using a funnel plot, however, as the number of studies was lower than 10, we did not report this following the Cochrane Handbook (19).

### Data Synthesis

After extracting the highest AUC and Standard Error, the random-effects model was used to calculate the pooled AUC. Heterogeneity was assessed using the Cochrane Q-test and I² statistic, where a p-value < 0.05 indicated statistically significant heterogeneity. Accordingly, I^2^ scores are divided into the following: moderate heterogeneity (30–60%), substantial heterogeneity (50–90%), or considerable heterogeneity (75–100%). The meta-analysis was executed by MedCalc Statistical Software version 19.2.6 (MedCalc Software bv, Ostend, Belgium; https://www.medcalc.org; 2020).

## Results

### Study Selection

**Figure 1:**
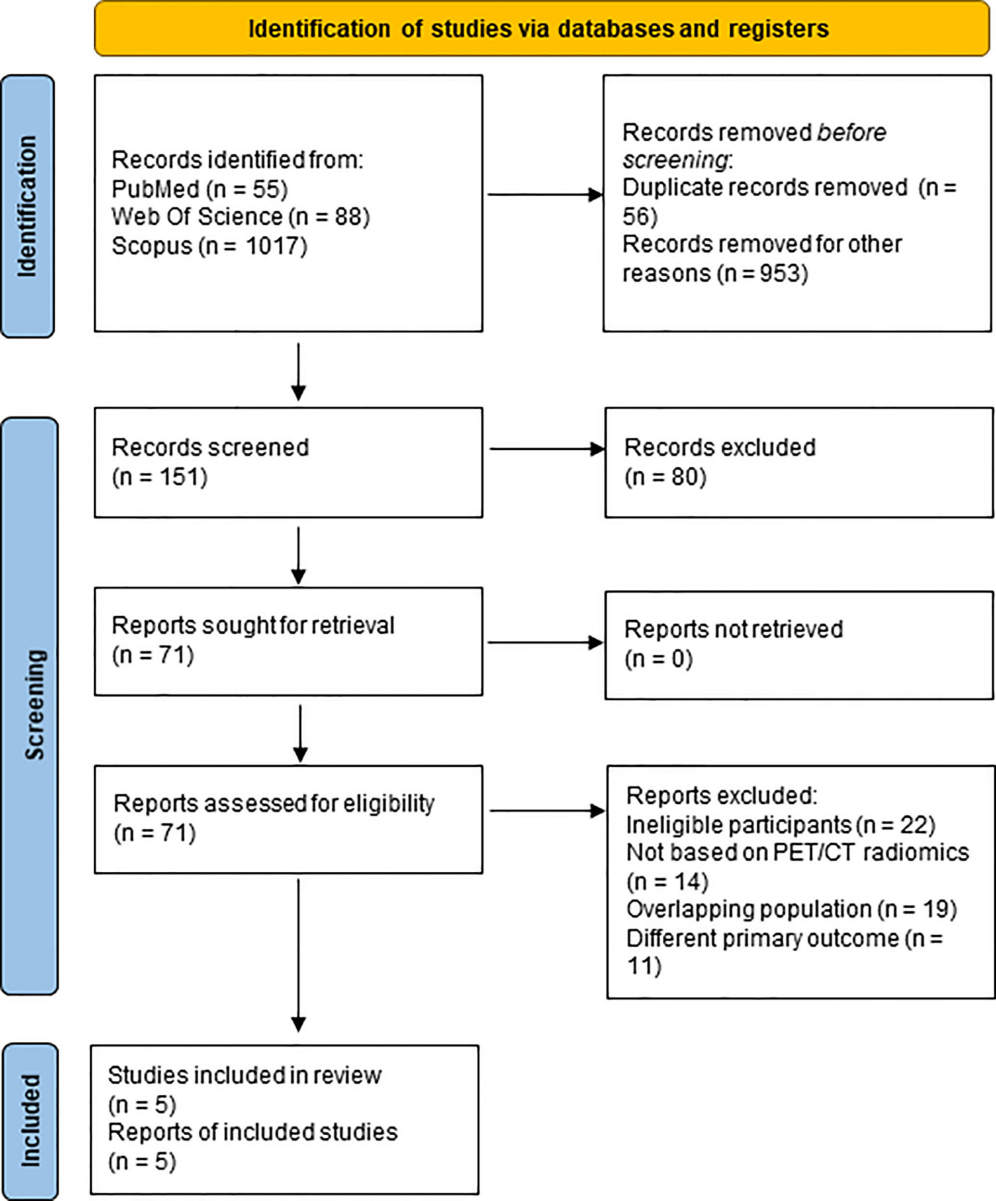
PRISMA Flow-chart.

A flow diagram of the search strategy results is presented in **Figure 1**. After removing 56 duplicates and 953 articles in different medical fields, 151 articles were obtained – all in English, from which 80 studies were excluded after examining their titles and abstracts. Subsequently, 71 studies were selected for full-text reading. Of these, 66 were excluded as they did not match the inclusion criteria or had an overlapping population. Finally, five studies were included in the present systematic review.

### Quality Assessment

Radiomics Quality Score (RQS) for each of the five included studies is reported in **Table 1**. RQS ranged between 5 (21) to 16 points (20). None of them reported a phantom study, imaging at multiple time points, biological correlates, cost-effectiveness analysis, nor a prospective study. All studies adequately recorded the feature reduction on adjustment for multiple testing and potential clinical utilities. The remaining items illustrated a strong level of heterogeneity among the selected group.

**Table 1:** Quality assessment – Radiomics Quality Score (RQS).

Criteria/Study	van Rossum, 2016 (20)	Yip, 2016 (21)	Beukinga, 2017 (22)	Rishi, 2020 (23)	Murakami, 2021 (24)
Image protocol quality	1	1	2	2	0
Multiple segmentation	1	0	1	1	1
Phantom study	0	0	0	0	0
Imaging at multiple time points	0	0	0	0	0
Feature reduction on adjustment for multiple testing	3	3	3	3	3
Multivariable analysis	1	0	1	0	0
Biological correlates	0	0	0	0	0
Cut-off analysis	0	1	0	1	1
Discrimination statistics	2	1	2	2	2
Calibration statistics	2	0	2	0	0
Prospective study	0	0	0	0	0
Validation	2	-5	2	-5	2
Comparison to ‘gold standard’	2	2	0	2	0
Potential clinical utility	2	2	2	2	2
Cost-effectiveness analysis	0	0	0	0	0
Opens science and data	0	0	0	0	3
**Total Score (max 36 points)**	**16**	**5**	**15**	**8**	**14**

### Review of Type of Radiomics Feature and Other Features in Selected Studies

According to the International Symposium on Biomedical Imaging (ISBI) standards, the radiomics features are divided into morphology class (e.g., shape-based), first-order class (e.g., histogram-based), and second-order class (e.g., texture-based).

One study used the morphology class feature (20) while three studies employed (21, 22, 24) the second-order class including different grey-level matrix (i.e., grey-level run-length matrix, grey-level co-occurrence matrix, grey-level size-zone matrix, grey-level dependence matrix); lastly, Rishi et al. (23) used both the first-order (i.e., intensity and shape) and second-order classes.

Four out of five studies used features selection methods for dimensionality reduction (19–22), while the fifth study identified six textures according to clinical values in prognostic and treatment response assessment after extracting textural features through complex mathematical models (18).

Among studies that adopted features selection methods, three adopted wrapper methods (20–22). In particular, Beukinga et al., after pre-selecting 144 of the 147 features, identified, through a univariable logistic regression analysis, 24 significant variables, subsequently used to develop six different models (21). Murakami et al. starting from 4250 features and adding 22 more features selected through the LASSO analysis and one chosen from the original image features, compared performances of five neural network models generated in 5-fold cross-validation steps (20). Rishi et al. determined the predictive model after building and validating four models using leave-one-out cross-validation on a total of 126 features and some composite features (22). Instead, Van Rossum et al., after using a univariable analysis from which many potential predictors were identified, used a filter approach based on a standardized pre-selection variables method according to the following three inclusion rules: (1) variables with p ≤ 0.25 in the univariable analysis; (2) variable with the lowest p-value in case of highly correlated pairs of variables; (3) features with an ICC ≤ 0.70 in the test-retest analysis.

### Study Characteristics

The authors provided a descriptive summary of the characteristics of the studies in **Table 2**.

**Table 2:** Studies’ Characteristics.

Author	Country	Data Source	Patients	Gender (Females/Males)	Age	Histology	Localisation	nCRT	Training Set	External Validation	Highest AUC	SE	Pathological response	Model
**van Rossum (** 20) **(2016)**	USA	Single-institution	217	15/202	PathCR: 58.8 ± 12.3; No pathCR: 60.1 ± 9.9	AC	Middle third: 3; Distal third: 195; GEJ: 19	45-50.4 Gy + 5FU with either a platinum compound or taxane	217	No	0.77	0.030	CR = 59	Multivariable Logistic regression with stepwise backward elimination
													No CR = 158	
**Yip** (21) **(2016)**	USA	Single-institution	54	10/44	65 yr	AC: 50; SCC: 4	NR	45-50.4 Gy + a platinum compound with either 5FU or taxane	45	No	0.65	0.100	CR=8	Kaplan – Meier with the log-rank test
													No CR = 37	
**Beukinga** (22) **(2017)**	Netherland	Single-institution database	97	15/82	< 70 yr: 78; ≥ 70 yr: 19	AC: 88; SCC: 9	Mid: 4; Distal: 62; GEJ: 31	41.4 Gy + carboplatin /paclitaxel	97	No	0.74	0.050	CR: 19 – No CR: 78	Logistic regression with LASSO
**Rishi** (23) **(2020)**	USA	Single Institution	68	21/47	65.3 yr (43–82)	NR	Upper: 3; Mid: 7; Distal: 34; GEJ: 24	45-56Gy+ 5FU and cisplatin	68	No	0.87	0.010	CR: 34	Kaplan- Meier
**Murakami** (24) **(2021)**	Japan	NR	98	15/83	66 yr (35–78)	NR	Upper: 22; Middle: 46; Lower GEJ: 30	40Gy + 5FU and cisplatin	98	Yes	0.95	0.004	CR: 44	Neural Network Classifier

CR, complete response; AC, Adenocarcinoma; SCC, Squamous Cell Carcinoma; nCRT, neoadjuvant chemoradiotherapy.

We included a total of five studies: three carried out in the USA (20, 21, 23), one in the Netherlands (22), and one in Japan (24).

One study included more than 200 patients (20), whereas four studies had less than 100 patients (21–24).

Altogether, 534 patients were included in this study (458 males and 76 females). The patients’ median age ranged between 35 years (24) and more than 80 years (23). Two of the selected papers did not report the patients’ histology, while the other three were primarily focused on adenocarcinoma patients (20–22).

All patients were treated with external beam radiation therapy and concurrent chemotherapy. A total radiation dose ranging from 40 to 50.4 Gy was delivered in daily fractions of 1.8–2Gy. Among the 534 patients analysed in the 5 studies, in the vast majority of cases RT was delivered with three-dimensional conformal radiation therapy (3D-CRT) and intensity-modulated radiation therapy (IMRT) (18–22), in a minority of cases (12%) proton therapy was performed (19). Concomitant chemotherapy generally consisted of a platinum compound with fluoropyrimidine or taxane.

### Meta-Analysis

**Figure 2:**
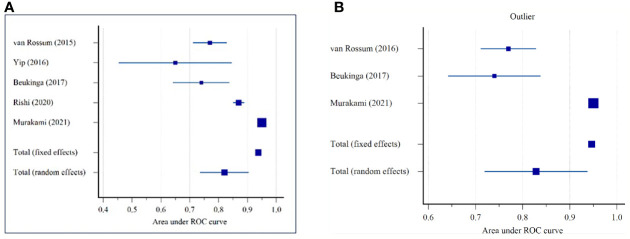
Forest plot for the area under the receiver operating characteristics (ROC) curve for predicting the pathological response in patients with oesophageal cancer: **(A)** All Sample (n = 5 studies); **(B)** Without small studies (n = 3 studies).

The pooled AUC for the five studies was 0.821 (95% CI: 0.737–0.904), according to the results from **Table 3** and **Figure 2A**. The I^2^ was 96.46% (95% CI: 94.00–97.92%) (Cochrane Q = 113.09, p < 0.0001), displaying a substantial heterogeneity among studies. After excluding the small studies (e.g., studies with less than 70 patients), AUC was 0.829 (95% CI: 0.719–0.938) (**Figure 2B**). The sensitivity analysis (fixed-effect model) depicted no significant differences from the previously reported results.

**Table 3:** Summary Table Meta-Analysis.

Study	ROC Area	Standard Error	95% CI	z	P	Weight (%)	
						Fixed	Random
van Rossum (2016) (20)	0.770	0.030	0.711 to 0.829			1.21	22.02
Yip (2016) (21)	0.650	0.100	0.454 to 0.846			0.11	10.51
Beukinga (2017) (22)	0.740	0.050	0.642 to 0.838			0.44	18.46
Rishi (2020) (23)	0.870	0.0100	0.850 to 0.890			10.92	24.36
Murakami (2021) (24)	0.950	0.0035	0.943 to 0.957			87.33	24.65
Total (fixed effects)	0.938	0.0033	0.931 to 0.944	283.859	<0.001	100.00	100.00
Total (random effects)	0.821	0.0428	0.737 to 0.904	19.186	<0.001	100.00	100.00

## Discussion

To the best of our knowledge, this is the first meta-analysis exclusively analysing the power of 18F-FDG PET-based radiomics to predict the pCR after neoadjuvant chemoradiotherapy in oesophageal and gastro-oesophageal junction cancer.

In oesophageal cancer, 18F-FDG PET/CT is part of the initial work-up improving the accuracy of the clinical staging and better assessing distant metastatic disease and is frequently incorporated into radiotherapy planning (25). Moreover, the prognostic value of 18-F-FDG PET in assessing pCR after neoadjuvant radiochemotherapy showed contrasting results (26). To predict treatment outcome is crucial in oesophageal and gastro-oesophageal junction cancer in order to select patients more likely to escalate or de-escalate therapy. Radiomics is an appealing field of research and is deeply under investigation.

Radiomics is an advanced method to extract imaging features and thereby quantify tumour phenotype from medical images (16). Using radiomics, a single medical image shows more information, and extraction and analysis of hundreds of imaging features can be obtained. In general, radiomics features are classified into morphological features (size and shape), first-, second-, and higher-order textures. As reported by Gillies et al., first-order statistics describe the distribution of values of individual voxels without concern for spatial relationships and are generally histogram-based methods. Second-order statistical descriptors generally are described as “texture” features and provide a measure of intratumoral heterogeneity. Higher-order statistical methods impose filter grids on the image to extract repetitive or non-repetitive patterns (12).

Among them, texture analyses depicting and objectively quantifying tumour heterogeneity seem to be of great interest in order to identify potentially responders and non-responders (9, 18). Moreover, these imaging features can be used in predictive modelling alone or with other patient-related data (e.g. clinical data, pathological data, and genomic data). This could lead to tailored and potentially most effective treatment for each patient (12, 18).

In this regard, among the studies here analysed only one reported the influence of clinical parameters on the probability of pCR. In particular, smaller tumour length based on endoscopic ultrasound and lower T stage (i.e. T2 *vs* T3) and negative post-treatment endoscopic biopsy significantly influence the probability of pCR (19).

Moving to our meta-analysis, our findings provided that the pooled AUC of the five selected studies was quite high at 0.821 (95% CI: 0.737–0.904) and not influenced by the studies’ sample size. Moreover, the I^2^ score was 96.46% (95% CI: 94.00–97.92%, p < 0.0001). Thus, substantial heterogeneity existed among the studies: this is explained by different image scanners and imaging elaboration, which influenced radiomics features (27).

The dissected studies’ RQS ranged from 5 (21) to 16 (20) – considered a poor-quality level because some items are not present. Although RQS is widely used in the quality assessment of radiomics studies, the low methodological quality is their main limit and comparable to most systematic reviews in other tumour sites (15, 28, 29). Although the quality was not always as desired, all studies included in this analysis deliver the most complete overview of the existing literature.

Overall, none of the analysed studies performed a cost-effectiveness analysis; they were not prospective, did not perform a phantom study or imaging at multiple time points, or had a biological correlation. Similarly, in a recent systematic review on nasopharyngeal tumours using MRI radiomics features, none of the included studies executed neither a phantom study nor a cost-effectiveness analysis (28). Conversely, all the studies addressed their potential clinical utility and used a feature reduction or adjustment for multiple testing. In a recent meta-analysis of renal cancer, most of the 57 studies reported a potential clinical utility, and only 51% employed a feature reduction (30).

In the future, the RQS principles should be considered before planning radiomics studies to ensure satisfactory quality. Although a high- or low-quality range was not stated in the RQS guidelines (18), a cut-off score of 30% should be planned as suggested by Wesdorp et al. (14).

Among the five studies included in our analysis, three enrolled a majority of adenocarcinoma 335/368 (20–22), while the remaining two (23, 24) did not report the histology of their 166 patients. Therefore, due to this heterogeneity, a stratification for histology (adenocarcinoma *vs*. squamous cell cancer) could not be performed in the present meta-analysis.

Neoadjuvant chemoradiotherapy followed by surgery is a well-established approach in oesophageal squamous cell carcinoma and gastro-oesophageal adenocarcinoma (4, 31), while definitive chemoradiotherapy is often preferred in cervical oesophageal cancer (32). The tide of active surveillance in cervical oesophageal cancer was also applied to thoracic oesophageal squamous cell carcinoma, providing a salvage surgery for persistent or recurrence disease (7, 33). This approach is also currently under investigation in gastro-oesophageal adenocarcinoma (5, 6). Future radiomics studies stratifying patients between squamous cell tumours and adenocarcinoma could be of great interest and grant further evidence for choosing optimal care.

The articles included in this review suggest that first- and second-order features contributed to the response assessment, predominantly in predicting pCR. Tumours with low heterogeneity were more likely to reach a pCR. In detail, van Rossum et al. developed a prediction model adding four comprehensive 18F-FDG PET texture/geometry features (i.e., baseline cluster shade, change in run percentage, change in co-occurrence matrix entropy, and post-radiation roundness) and improved the AUC to 0.77 instead of the 0.67 obtained with clinical models (20). A tumour exhibiting a heterogeneous 18F-FDG PET distribution – baseline cluster shade – was less likely to reach pCR in their analysis on 217 oesophageal adenocarcinoma cancer patients (19). Beukinga et al. depicted a model that combined the clinical T-stage and 18F-FDG PET-derived textural feature long run low gray-level emphasis. After internal validation, the model provided high accuracy in predicting pCR with an AUC score of 0.74 (22). However, both authors concluded that their results did not translate into a clinically relevant benefit. In Yip et al., the change in the run-length and size-zone matrix textures significantly differentiated non-responders from partial and complete responders (AUC = 0.65) (21).

More recently, Murakami et al. constructed a predictive model with a good AUC score of 0.95, extracting 22 second-order radiomics features (24). Lastly, Rishi et al. illustrated that a composite model (based on PET and CT) improved pCR predicting power with an AUC score of 0.87 (23).

As reported in literature and in our findings, tumour heterogeneity seems to have impact on tumour response, since tumours with greater intratumoral heterogeneity are often assumed to have an aggressive biology (34). However, these results are not definitive due to the lack of standardized methodology in extracting and analysing radiomics features. Among the studies here analysed, Beukinga et al. showed that the most predictive textural features were LRLGLe-PET and RP-CT. Both were higher in complete responders, corresponding to homogeneous 18F-FDG uptake. A possible explanation of homogeneity and heterogeneity is hypoxia and necrosis (21).

Overall, radiomics features could objectively and quantitatively describe distinctive tumour “radio-phenotypes”. In future, all these radio-phenotypes could potentially substitute a “real biopsy” and explain treatment sensibility or resistance describing and identifying metabolic activity, proliferation grade, angiogenesis as well as genomic stability or instability (22).

Remarkably, we discovered that the pooled AUC after excluding two small studies with less than 70 patients remains pretty high at 0.829 (95% CI: 0.719–0.938). A recent review underlined the relevance of the sample size to allow high dimensional models and machine learning approaches to be statistically robust considering an adequate cut-off > 100 or > 200 patients. The performance of the existing algorithm would be applied to new large datasets (35). In the present review, one study enrolled more than 200 patients (20), whereas two approached 100 (22, 24).

A machine-learning algorithm was used in four out of the five studies (20, 22–24). In the last few years, the machine learning approach has been widely used. Interestingly, the most recent study by Murakami et al. used a neural network classifier to construct their prediction model (24). Deep learning is a subfield of machine learning, rapidly gaining interest among the radiation-oncology community; it may offer a better model complexity; however, the published literature on tumour response prediction is relatively scarce and requires a much larger sample size (15, 36).

Despite the encouraging findings of the present meta-analysis in using 18F-FDG PET/CT radiomics to predict treatment responses in oesophageal cancer, some limits should be underlined. First, few studies were included; thus, publication bias analysis was not performed because it was not appropriate. Second, all included studies were retrospective and performed in a monocentric setting. Third, demographic heterogeneities were observed among studies due to different race ethnicity. Furthermore, they used different PET scanners, and the selection of the features was based on different methodologies, distinct methods of tumour volume segmentation (manual delineation and semi-automatic segmentation), and often on differing in-house software. An additional limitation of these studies is that their authors focused on different feature sets, and the data analysis and interpretation were based on several approaches. Moreover, only one study (19) reported clinical characteristics between pCR and non pCR groups. Lastly, they differed in treatment schedules in terms of radiation dose and chemotherapy schedule. Among the five studies, only Beukinga et al. analysed patients who underwent the CROSS schedule, considered the standard of care in a neoadjuvant setting (21).

Based on these results, we conclude that 18F-FDG PET/CT-based radiomics images have a high accuracy in predicting pCR to neoadjuvant chemoradiotherapy. Overall, the main concern is reaching higher data quality in oesophageal and gastro-oesophageal junction cancer. Next step is to plan studies incorporating quality control. Future research should focus on developing predictive models, through well-designed and appropriately powered prospective studies, with the aim to complement the current clinical findings with radiomics, and further stratify and personalise oncologic treatment.

## Data Availability Statement

The original contributions presented in the study are included in the article/supplementary material. Further inquiries can be directed to the corresponding author.

## Author Contributions

LD, MG, and SD contributed to conception and design of the study. MG performed the statistical analysis. LD and MG wrote the first draft of the manuscript. SD and AV wrote the discussion. GP and SC helped collect literature and participated in discussions. DL, MV, DLR, MC, and MCP examined and verified the results. All authors read and approved the final manuscript.

## Conflict of Interest

The authors declare that the research was conducted in the absence of any commercial or financial relationships that could be construed as a potential conflict of interest.

## Publisher’s Note

All claims expressed in this article are solely those of the authors and do not necessarily represent those of their affiliated organizations, or those of the publisher, the editors and the reviewers. Any product that may be evaluated in this article, or claim that may be made by its manufacturer, is not guaranteed or endorsed by the publisher.
